# Analysis of HPV distribution in patients with cervical precancerous lesions in Western China

**DOI:** 10.1097/MD.0000000000007304

**Published:** 2017-07-21

**Authors:** Kemin Li, Rutie Yin, Qingli Li, Danqing Wang

**Affiliations:** a The Department of Obstetrics and Gynecology, West China Second University Hospital, Sichuan University, Chengdu; b Key Laboratory of Birth Defects and Related Diseases of Women and Children (Sichuan University), Ministry of Education, China.

**Keywords:** cervical precancerous lesions, HPV distribution, Western China

## Abstract

Cervical human papillomavirus (HPV) infection is a dangerous disease, whose subtypes exhibit different distribution patterns in particular countries, regions, and races. In this study, the HPV distribution in patients with cervical precancerous lesions in Western China was investigated to assess a probability for prevention of cervical cancer and the clinical application of an HPV vaccine in China. The retrospective study of patients with different HPV subtypes and cervical precancerous lesions, who have undergone loop eelectrosurgical excision procedure, cold knife conization, or a total hysterectomy in our hospital from January 2016 to August 2016, was performed. All patients were tested for 27 HPV subtypes via the liquid suspension chip technology (Luminex 200). A total of 3393 cases of cervical intraepithelial neoplasia (CIN) were investigated, including 1098 cases of CIN I, 762 cases of CIN II, and 1533 cases of CIN III. The overall HPV infection rate was 82.58%. The high-risk HPV infection rate was 76.61%, and the low-risk rate was 9.88%. The most common 5 subtypes were HPV16, HPV52, HPV58, HPV33, and HPV18. The patients were grouped into 6 age groups: ≤20, 21 to 30, 31 to 40, 41 to 50, 50 to 64, and ≥65. The HPV subtypes’ distribution varied across different age groups. In patients with cervical precancerous lesions in Western China, the top 5 HPV subtypes with the highest infection rates were HPV16, HPV52, HPV58, HPV33, and HPV18. The rate of cervical precancerous lesions unrelated to HPV was 17.42%. Thus, HPV screening with no cytology may leave unobserved about 20% of cervical precancerous lesions, which is worth of significant clinical attention.

## 1 Introduction

Cervical cancer is a serious threat to female health. In 2015, there were 98,900 new cases of cervical cancer and 30,500 cervical cancer-related deaths in China.^[1]^ The global annual incidence of cervical cancer in 2012 was 528,000, the annual death rate was 266,000. It is the fourth most common cancer in women worldwide.^[2]^

Cervical intraepithelial neoplasia (CIN) is a one-way pathophysiological process with 2 distinct outcomes. The first one is a spontaneous regression of the cervical lesions after evolution, which, in general, does not develop into cervical invasive carcinoma. The second outcome is the potential occurrence of cancer during the development of cervical lesions: if not timely screened or treated properly at early stages, it may develop into invasive carcinoma. Studies have shown that the probability for CIN I, CIN II, and CIN III to develop into cervical cancer are15%, 30%, and 45%, respectively, and occasionally, CIN I and CIN II may directly develop into cervical invasive carcinoma without turning into CIN III first.^[3]^ In general, it takes approximately 10 to 20 years of precancerous lesions before cervical cancer develops. Therefore, standard and reasonable early screening and early treatment may effectively reduce the incidence of cervical cancer and improve survival and life quality for thousands of female patients.

The main prerequisite for development of cervical cancer and precancerous cervical lesions is persistent infection with high-risk human papillomavirus (HPV).^[4–6]^ As HPV infection rates and distribution of different HPV types are known to vary significantly in different countries and regions,^[6–12]^ their investigation in specific areas is expedient for providing a scientific basis for the measures and methods aimed at distribution minimization and more effective treatment of cervical cancer, including the development of an HPV vaccine. Thus, the results obtained would be instrumental for developing more effective prevention and control strategies for the regional cervical cancer treatment. Cervical cytology and HPV tests are widely used for cervical cancer screening and can effectively reduce its incidence. However, the incidence of cervical cancer in China exhibits an increase and shifts to younger age groups. This can be attributed to the fact that China is a developing country, whose citizens are not fully aware of the importance of screening for cervical cancer, and many areas do not yet have universal cervical cancer screening. In this study, the HPV subtypes in patients with cervical precancerous lesions, who were treated in the West China Second University Hospital of Sichuan University (the largest gynecological cancer center in Western China) from January 2016 to August 2016, were retrospectively analyzed.

## 2 Materials and methods

### 2.1 Materials

The study subjects were patients with cervical precancerous lesions, who were treated in our hospital from January 2016 to August 2016, regardless of age. These patients have undergone loop eelectrosurgical excision procedure, cold knife conization, or a total hysterectomy. Any patients with a medication treatment history for HPV infection, with cervical surgery and/or hysterectomy history, with liver, kidney, or systemic diseases, and any patients treated with vaginal medication within a week were excluded from the analysis.

### 2.2 Methods

The retrospective studies were conducted, where each patient was placed in the lithotomy position and sterile cotton swabs were used to clean mucus. A dry, sterile cotton swab was inserted into the cervix and rotated clockwise for 5 to 8 circles, and the exfoliated cervical cells were stored in a cell preservation solution. A kit [Qiagen RNA/deoxyribonucleic acid (DNA) Mini kit; TELLGEN Life Science Co. Ltd., Shanghai, China] for purifying DNA from it produced by Qiagen was used to extract DNA from the specimens. Afterward, the extracted DNA was dissolved in 50 μL of elution buffer and stored at −20 °C for use. The purified DNA was placed in the genotyping kit, namely, the HPV nucleic acid typing detection kit (flow fluorescence hybridization) produced by Qiagen, including 17 high-risk types (HPV-16, 18, 26, 31, 33, 35, 39, 45, 51, 52, 53, 56, 58, 59, 66, 68, and 82) and 10 low-risk types (HPV-6, 11, 40, 42, 43, 44, 55, 61, 81, and 83). The hybridization products were tested by using a Luminex 200 (Luminex Corporation, Texas), which is a multifunctional suspension array system.

All patients underwent a colposcopy, and a cervical biopsy was performed. Two independent pathology specialists reviewed the paraffin-embedded tissue sections of cervical precancerous lesions. Cervical histopathological examination results were grouped into the following grades: CIN I, CIN I or worse (CIN I^+^), CIN II, CIN II or worse (CIN II^+^), CIN III, or CIN III or worse (CIN III^+^). Any patients with negative or invasive cervical cancer were excluded from the study. As this study is retrospective, no ethical committee approval was required for its conduction, which is in compliance with the institutional and national policies concerning research approvals.

### 2.3 Statistical analysis

The SPSS 22.0 statistical software (SPSS Inc., Chicago) was used for the data analysis. The measurement data were represented as x ± s (mean ± standard deviation) and analyzed with the *t* test. The count data were expressed as rate and were analyzed with the χ^2^ test. The *P* value was used to indicate the significance; the test level was α = 0.05, and *P* < .05 was taken as statistically significant.

## 3 Results

### 3.1 Basic characteristics of the subjects

The basic characteristics of the research subjects are shown in Table 1. A total of 6152 patients with cervical precancerous lesions were initially included, and among these 1782 cases were excluded due to a history of cervical HPV infection and treatment, 732 cases were excluded due to a history of cervical surgery or treatment, 229 cases were excluded due to hysterectomy, and 16 cases were excluded due to liver, kidney, or systemic diseases. Thus, the final study covered 3393 patients with an average age of 39.4 years, and included 1098 cases of CIN I, 762 cases of CIN II, and 1533 cases of CIN III.

**Table 1:**
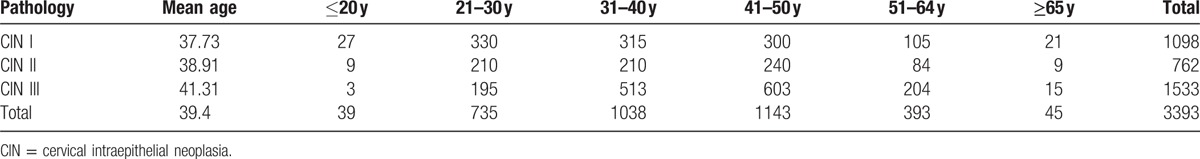
The characteristic of 3393 CIN patients.

### 3.2 Distribution of HPV subtypes in patients with CIN

**Figure 1:**
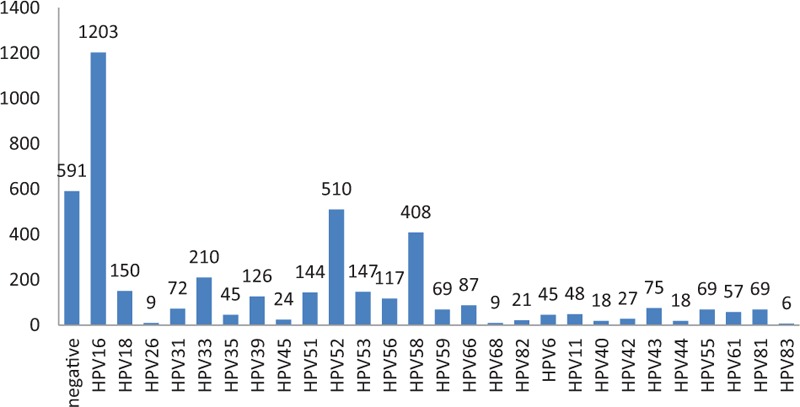
The distribution of human papillomavirus subtypes in cervical intraepithelial neoplasia patients.

**Figure 2:**
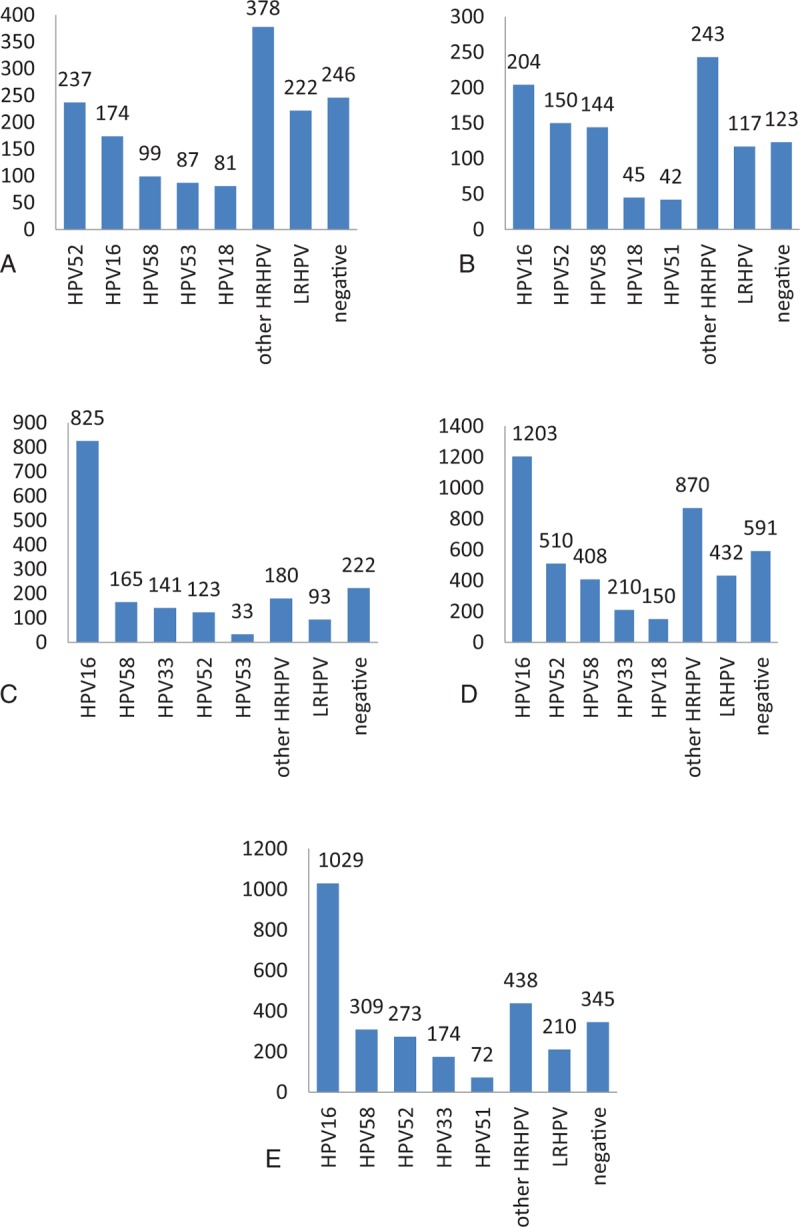
(A) The distribution of human papillomavirus (HPV) subtypes in cervical intraepithelial neoplasia (CIN I) patients, (B) the distribution of HPV subtypes in CIN II patients, (C) the distribution of HPV subtypes in CIN III patients, (D) the distribution of HPV subtypes in CIN I+ patients, and (E) the distribution of HPV subtypes in CIN II+ patients.

The distributions of HPV subtypes in patients with CIN are tabulated in Table 2 and depicted in Figs. 1 and 2. For the total population of patients, the overall HPV infection rate was 82.58%, while those for high- and low-risk HPV groups were 76.61% and 9.88%, respectively. The top 5 subtypes with the highest infection rates were HPV16, HPV52, HPV58, HPV33, and HPV18, and 17.42% of the patients were HPV-negative. Among the CIN II+ patients, the HPV infection rate was 84.97%, and the most common 5 subtypes were the same: HPV16, HPV52, HPV58, HPV33, and HPV18. Among these patients, 15.03% of the patients were HPV-negative.

**Table 2:**
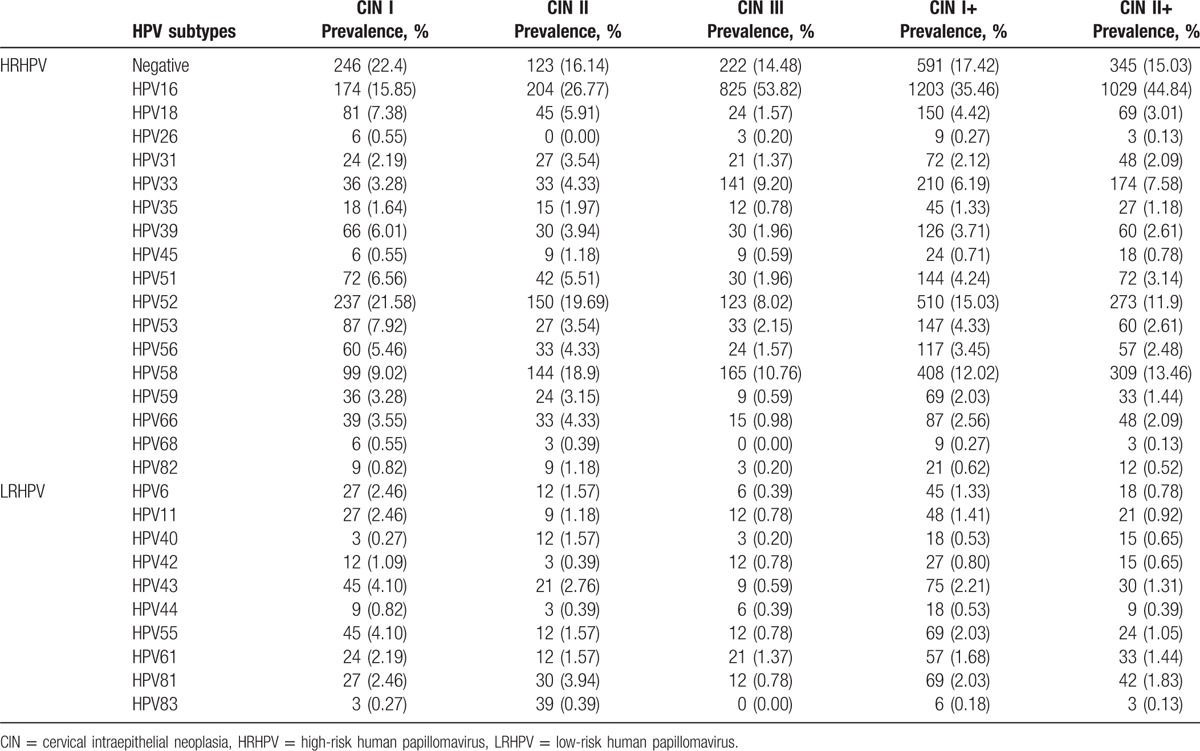
The distribution of HPV subtypes in CIN patients.

### 3.3 Distribution of HPV subtypes in patients from different age groups

**Figure 3:**
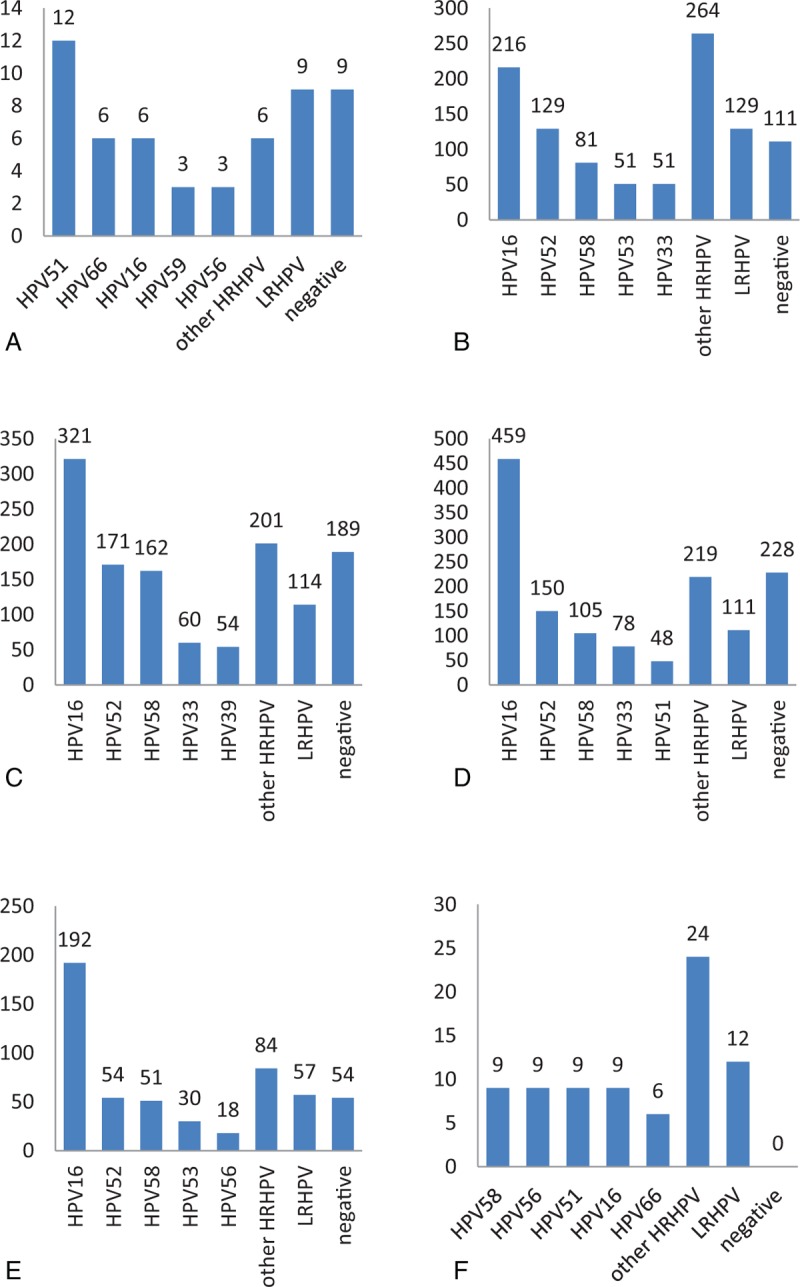
The distribution of human papillomavirus subtypes in different age group in cervical intraepithelial neoplasia patients (A: ≤20 years, B: 21–30 years, C: 31–40 years, D: 41–50 years, E: 51–64 years, and F: ≥65 years).

The distribution of HPV subtypes among patients from different age groups is presented in Tables 3–5 and depicted in Fig. 3. The highest HPV infection rate was 100% for patients over 65 years of age, while the lowest infection rate was 76.92% for patients of age ≤20. Thus, the distribution of HPV subtypes differed for various age groups.

**Table 3:**
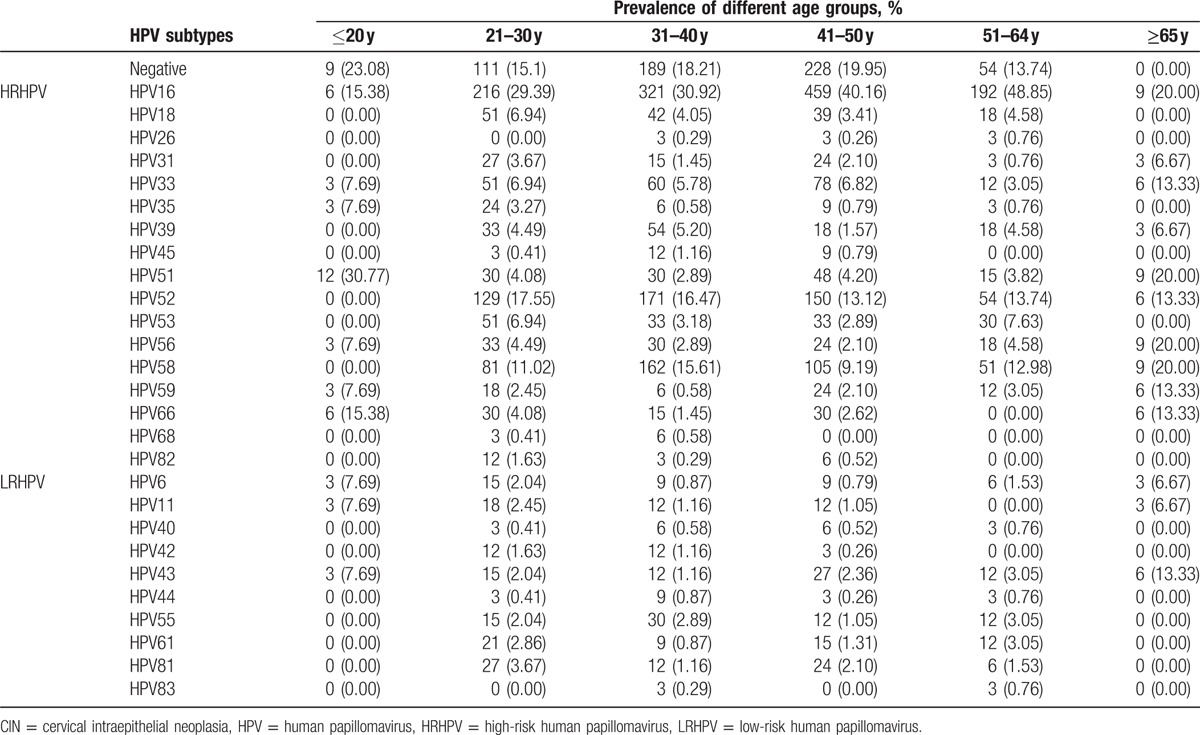
The distribution of HPV subtypes in CIN I+ patients.

**Table 4:**
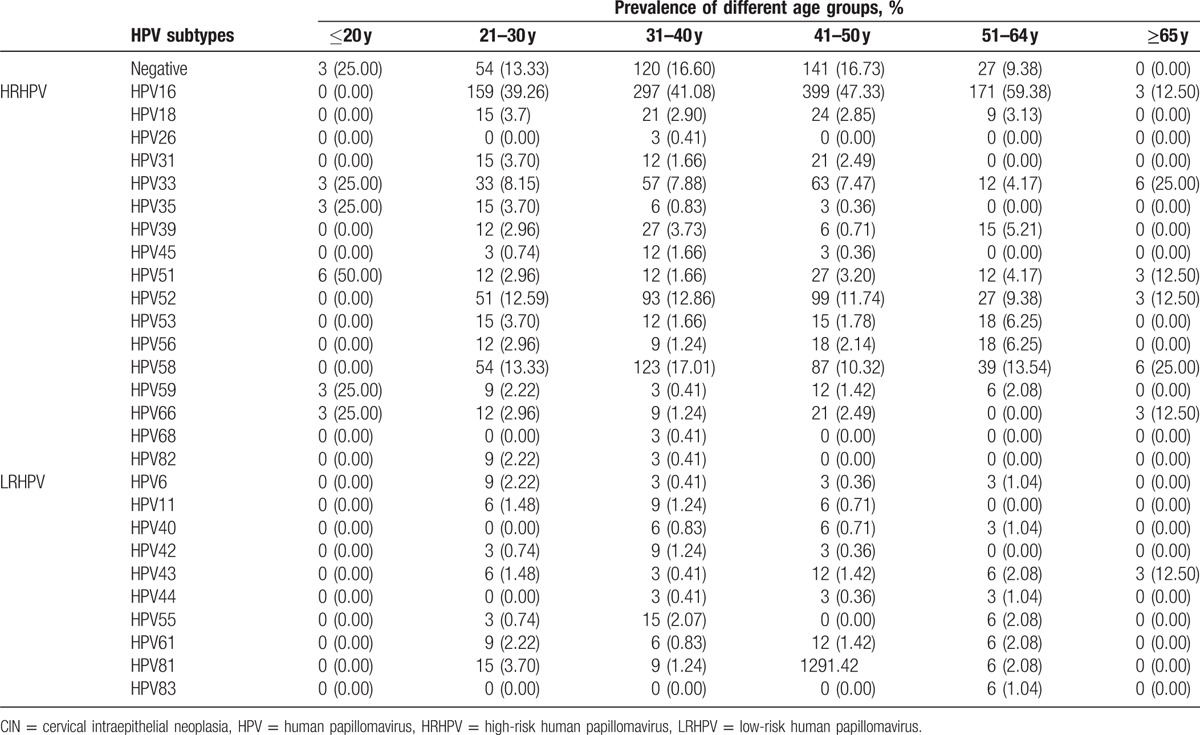
The distribution of HPV subtypes in CIN II+ patients.

**Table 5:**
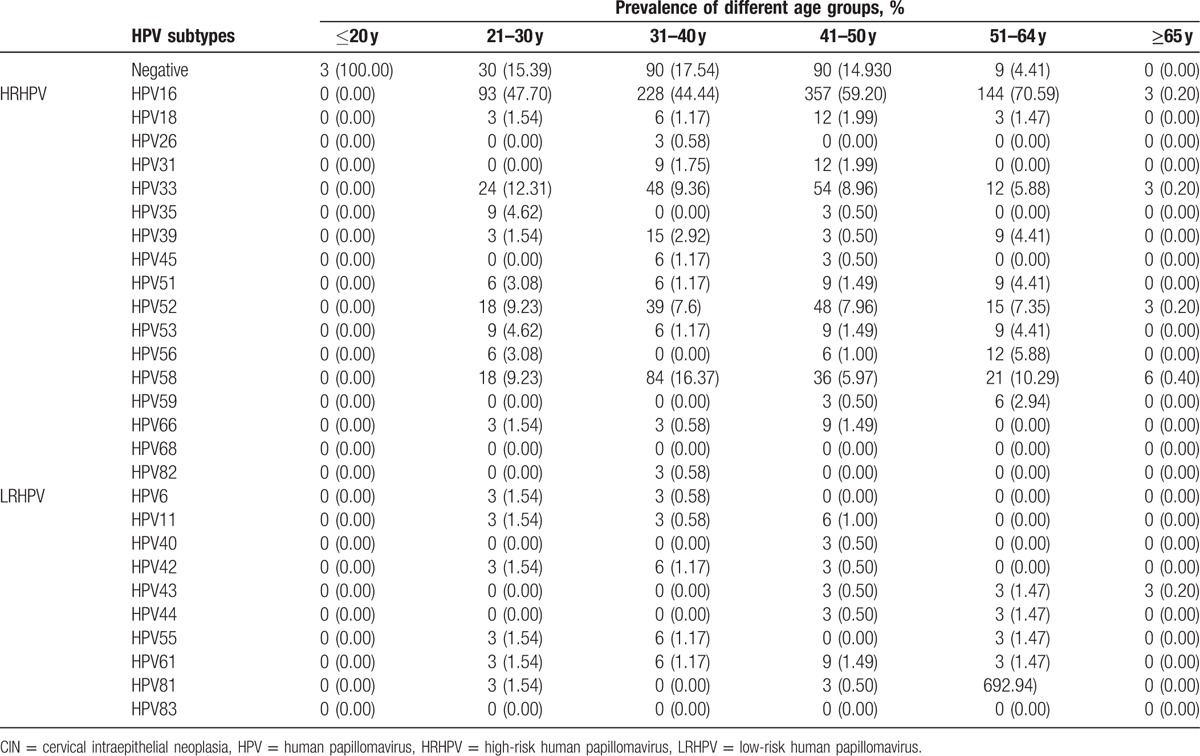
The distribution of HPV subtypes in CIN III patients.

Among the CIN II+ patients, the HPV infection was highest among patients of age ≥65, with a value of 100%; the most common 5 subtypes were HPV33, HPV58, HPV16, HPV51, and HPV52, a indication which holds for all patients. The lowest infection rate was for patients of age ≤20, with a value of 75%, and the most common 5 subtypes were HPV51, HPV33, HPV55, HPV59, and HPV66, which was also relevant for all patients. The distribution of HPV subtypes exhibited a variation among age groups.

### 3.4 HPV infections with multiple subtypes

**Figure 4:**
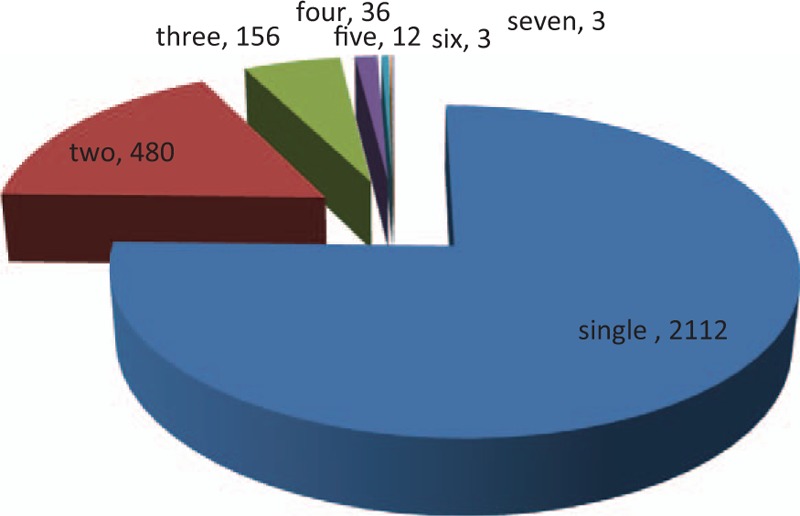
Multiple infections of human papillomavirus subtypes in cervical intraepithelial neoplasia patients.

The multiple infections of HPV subtypes are tabulated in Table 6 and depicted in Fig. 4. Most patients were infected with a single HPV subtype, with an infection rate of 62.25% (2112/3393). Among the patients infected with multiple subtypes, a double infection was most common, with percentage14.15% (480/3393). The largest number of HPV subtypes revealed in a single patient in this retrospective study was equal to 7, of which cases were observed only in 3 patients (0.09%).

**Table 6:**
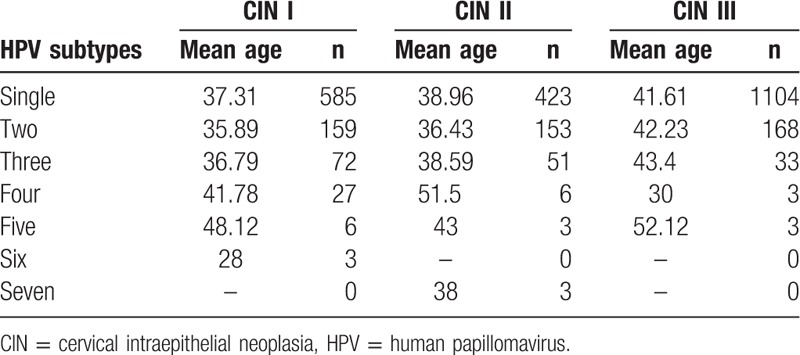
Multiple infections of HPV subtypes in CIN patients.

## 4 Discussion

Persistent infection with high-risk HPV is a prerequisite for the occurrence and development of cervical cancer and precancerous lesions in women. The distribution of HPV subtypes varies across different countries and even across different regions of a country, and the cervical precancerous lesions and cervical cancer related subtypes are different as well. Therefore, an analysis of the HPV subtypes of different ethnic groups in a country or a region is critical for the prognosis evaluation, as well as for making review plans for outpatients, clinical treatment plans for inpatients, and developing preventive and therapeutic HPV vaccines.

Studies have shown that the overall HPV infection rate is 38.1% in the Henan area, and HPV16 was the most common 1 among all subtypes, with a value of 8.9%.^[13]^ A study conducted by Xiao et al^[14]^ on HPV infections in patients with cervical precancerous lesions in the Beijing area demonstrated that the high-risk HPV infection rates are 38.1% and 32.4%, respectively, for CIN I and CIN II+ patients, and the most prevalent HPV subtypes for CIN I patients were HPV16, HPV58, HPV52, and HPV18. In contrast, the most prevalent ones for CIN II+ patients were HPV16, HPV58, HPV33, and HPV52. The reports on HPV infection in patients with CIN in the Western China are scarce, and the sample sizes of these studies are nonrepresentative.^[15–17]^ Thus, to develop reasonable screening and treatment plans for patients with HPV infections in the Western China, as well as provide evidence for the development of preventive and therapeutic HPV vaccines, we treated patients with cervical precancerous lesions admitted in our hospital from January 2016 to August 2016 as our study subjects and analyzed their HPV infection subtypes. The study demonstrated the following results: the overall HPV infection rate was 82.58%, and the high-risk HPV infection rate was 76.61%. The most common 5 subtypes were HPV16, HPV52, HPV58, HPV33, and HPV18, and 17.42% of the patients were HPV negative. Among the CIN II+ patients, the HPV infection rate was 84.97%, and the most common 5 subtypes were the same as for all patients, and 15.03% of the patients were HPV-negative. Except for the fact that HPV16 had the highest infection rate, the infection rates of other subtypes were quite different.

The HPV infections were different for patients of different age groups.^[18–20]^ Our study showed higher HPV infection rates among women over 40 years of age, and lower one for younger ones. This result is consistent with the high incidence of cervical cancer in women aged 45 to 60 in China. Therefore, it has become particularly important to disseminate information about cervical diseases in elder women, as well as perform HPV infection screening for this age group.

Previous studies have revealed that 80% of women have been infected with HPV throughout their lives; nevertheless, most of them were transient infections. The virus can be cleared by autoimmune mechanisms, usually in 8 to 24 months after infection. Only a small number of patients suffer from a persistent viral infection that further develops into lesions.^[21]^ In addition, the human immune system may eventually clear the virus, and clinically, the HPV test result would be negative, even if cervical lesions have formed by that time. HPV has proven to be the primary cause of cervical lesions, but it is not the only one. The occurrence and development of cervical lesions is the result of multiple factors. In this study, 17.42% of patients were HPV-negative, and 15.03% of patients with CIN II+ were HPV-negative.

In summary, the top 5 HPV subtypes with the highest infection rates in cervical precancerous lesions patients in the Western China were HPV16, HPV52, HPV58, HPV33, and HPV18. The HPV infection and its distribution varied across different age groups. Some patients with cervical precancerous lesions could not be detected through HPV screening. Therefore, solo HPV screening without cytology may miss approximately 20% of patients with cervical precancerous lesions, which requires additional clinical attention. This particular research effort being a single-center and retrospective study with a small sample size, its results should be confirmed and generalized by further large-sample, multicenter, prospective studies.
